# A genetic risk factor for idiopathic normal pressure hydrocephalus (iNPH).

**DOI:** 10.1186/2045-8118-12-S1-O51

**Published:** 2015-09-18

**Authors:** Takeo Kato, Hidenori Sato, Yoshimi Takahashi

**Affiliations:** 1 Yamagata University School of Medicine, Japan

## Introduction

Idiopathic normal pressure hydrocephalus (iNPH) is clinically important as a treatable gait disturbance and/or preventable dementia by shunt operation. iNPH usually occurs as a sporadic onset; however, genetic factor(s) is suggested to be involved in the pathogenesis of iNPH because of the presence of familial onset (two or more patients with NPH in a single family) of the disease, whose clinical and brain MRI features are indistinguishable from those of iNPH. Up to the present time, the genetic risk factor(s) for iNPH remains undetermined.

## Methods and Results

We made a whole-genome analysis for copy number variations (CNV), using DNA from peripheral blood of 8 subjects with MRI-supported possible iNPH/AVIM and 110 healthy controls. As a result, 4 (50%) of the 8 subjects were found to have a segmental copy number loss within the SFMBT1 (Scm-like with four MBT domains protein 1) gene. The copy number loss was heterozygous, and occurred at the 12 kb region within intron 2 of the SFMBT1 gene. Such genetic change was detected in one (0.9%) of the 110 controls. Next, using qPCR, we examined 8 patients with definite iNPH, who were shunt-responsive, and found that five (63%) of the 8 patients had a copy number loss at the same locus of the SFMBT1 gene. Immunohistochemical examination of the normal human brain revealed that the SFMBT1 protein localized mainly in the arterial walls, the ependymal cells, and the epithelium of the choroid plexus, which play a crucial role in the secretion, flow, and absorption of cerebrospinal fluid.

## Conclusions

The present study suggests that a copy number loss within the SFMBT1 gene may be a genetic risk factor for iNPH. Further studies on SFMBT1 will contribute to the elucidation of molecular basis of iNPH.

